# Interaction exposure effects of multiple disturbances: plant population resilience to ungulate grazing is reduced by creation of canopy gaps

**DOI:** 10.1038/s41598-020-58672-6

**Published:** 2020-02-04

**Authors:** Yushin Shinoda, Munemitsu Akasaka

**Affiliations:** 1 United Graduate School of Agricultural Science, Tokyo University of Agriculture and Technology, Fuchu, Tokyo, 183-8509 Japan; 2 Institute of Agriculture, Tokyo University of Agriculture and Technology, Fuchu, Tokyo, 183-8509 Japan

**Keywords:** Community ecology, Forest ecology

## Abstract

The impact of multiple disturbances on populations could be synergistic or antagonistic via disturbance interaction and are considered to be provoked by alternation of the impact of an ecosystem disturbance due to the effect of a preceding disturbance. The impact of a focal disturbance can also change when a preceding disturbance alters the proportion of individuals in a population exposed to these disturbances (i.e., interaction exposure effects), although this effect has not been addressed to date. Herein, we propose and test interaction exposure effects by elucidating disturbance interactions between canopy gap formation and ungulate grazing. Based on a vegetation and seed bank survey conducted on an island in Hokkaido, northern Japan, we examined whether canopy openness changes the impact of ungulate grazing on the occurrence probability of palatable plant species through the facilitation of germination. Species occurrence in the seed bank significantly decreased with increasing canopy openness under the presence of grazing; however, it slightly increased under the absence of grazing, suggesting that gap creation, which facilitates germination, exposes the seed bank to ungulate grazing. Because disturbances of various types often modify the habitat structure, these proposed disturbance interactions are expected to operate within various ecosystems and taxa.

## Introduction

An understanding of the impacts of disturbances on ecosystems is indispensable for comprehending the persistence of populations^1–3^. Climate change and increasing pressures from human development have increased disturbance frequency; therefore, it is more likely that ecosystems will be exposed to multiple co-occurring disturbances^4,5^ that could severely impact these environments^6–8^. Therefore, the interacting effects of multiple disturbances on populations should be understood to help predict the fate of ecosystems^4,9^.

**Figure 1:**
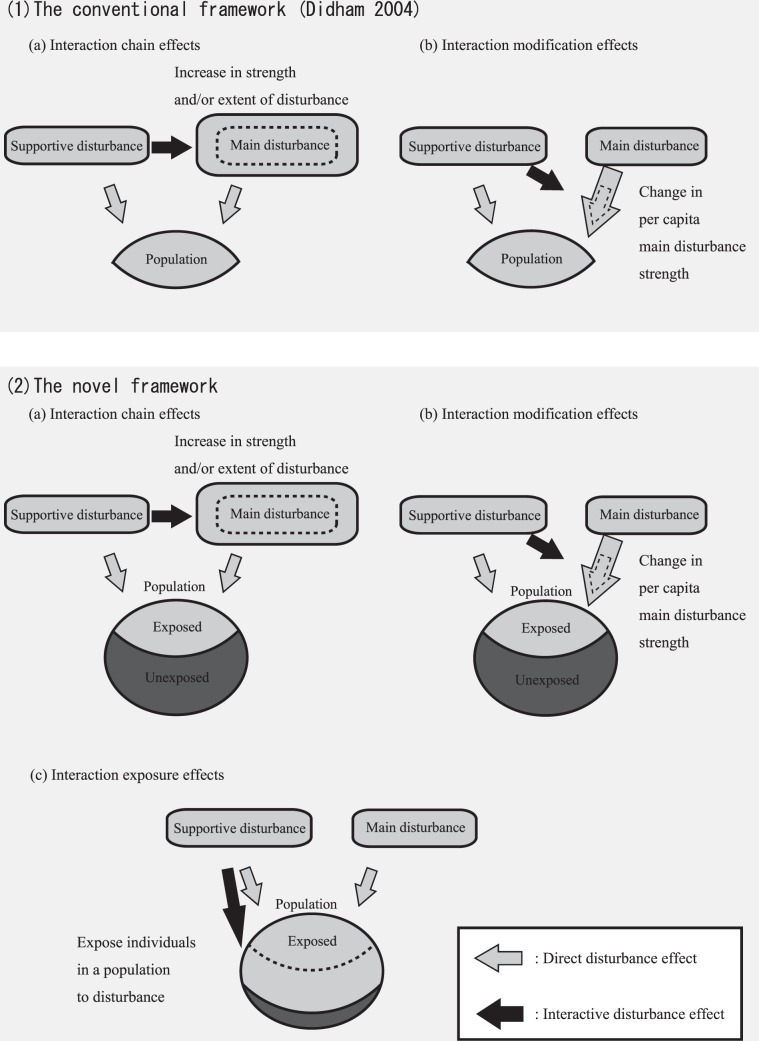
Conceptual diagrams of multiple disturbance interactions.

The population impact of multiple disturbances may be larger or smaller than the sum of the impacts of respective individual disturbances^4,10–14^. These cumulative or antagonistic impacts have been attributed to two types of interaction effects: interaction chain effects (linked disturbances in the context of Buma *et al*.^5^) and interaction modification effects^4,9^. Within the interaction chain effects, the total impact of disturbances on a population is altered by the indirect effects of a supportive disturbance to the *strength and/or extent* of main disturbances^15–17^ (Fig. 1a), e.g., a typical example of an interaction chain effect would be enhanced severity of a forest fire within a severely blown-down forest^16^. Within interaction modification effects, indirect effect(s) of supportive disturbance(s) on main disturbances modifies the per capita impact of the main disturbance^18,19^ (Fig. 1b). An example of interaction modification effects is that of savanna fires reducing the resistance of tree trunks to hurricanes, as rapid tree growth following fires results in low wood density^18^. Although both interaction chain and modification effects can reasonably explain the variations within disturbances’ impact on a population, we aim to consider the effect of disturbance interactions on population resilience^20,21^, which could be influenced by changes in the proportion of individuals within a population that are exposed to a disturbance due to disturbance interactions. In reality, not every individual in a population is necessarily subjected to a disturbance, as some could be protected within refugia or the seed bank^22,23^. However, a supportive disturbance could alter the proportion of individuals in a population that are exposed to main disturbances, here we use the term “interaction exposure effects” to describe this phenomenon (Fig. 1c). Individuals that avoid disturbance play a vital role in subsequent population resilience^24,25^. Consequently, multiple disturbances could dramatically reduce or increase population resilience through interaction exposure effects as compared to respective individual disturbances. Thus, disturbance interactions currently regarded as interaction modification effects would perhaps in some cases be better classified as interaction exposure effects. Discriminating between interaction exposure and modification effects could help to improve our understanding of these impacts on populations, species richness, and communities within the context of co-occurring multiple disturbances.

Disturbances may not only fatally damage plants but can also restrict their growth and reproduction (e.g., grazing, foraging, fire, or drought). Some species retain viable seeds in the soil serving as a seed bank; therefore, a fraction of these plant populations can escape exposure to disturbances via this protection^25–27^. Conversely, some disturbances can create conditions suitable for germination (e.g., typhoons generating canopy gaps or tip-up mounds and soil warming) and hence trigger germination within the seed bank^28–30^. Here, we consider canopy gap formation as a supportive disturbance and ungulate grazing as a main disturbance. These two disturbances could induce interaction exposure effects, whereby canopy gap formation facilitates germination from the seed bank by creating suitable conditions and plants are then exposed to the main disturbance of ungulate grazing. Consequently, interaction(s) between these two disturbances could reduce seed density in the bank to a greater extent than ungulate grazing alone. Furthermore, when disturbances continuously affect the plant population, the existing seed bank density would reduce dramatically due to cumulative impact. Therefore, interaction exposure effects from these disturbances may decrease population resilience to a greater extent than the sum of respective disturbances.

Didham *et al*.^4^ organized the types of disturbance interactions to explore the mechanisms causing synergistic or antagonistic effects by identifying the form and pathway of disturbance interactions. Their framework strongly promotes a systematic understanding of disturbance effects on population dynamics and improving system management; here we aimed to expand and modify this framework. In a similar vein, Buma^5^ and related research (e.g., Buma *et al*.^31^ and Cannon *et al*.^21^) classified variations of multiple disturbances based on whether the interaction affects resilience or resistance of a population (i.e., linked and compound disturbance framework). However, this dealt with both the synergistic and additive effects of multiple disturbances on resilience or resistance^5,16^, and did not focused on identifying the form or pathway of disturbance interactions. We therefore did not include this framework to our focus.

Our aim was to propose and verify the existence of what we describe as interaction exposure effects. To test this in the field, we focused on a forest ecosystem exposed to both ungulate grazing and canopy gap formation. We used plant species occurrence as a surrogate measure of the plant population’s response to disturbances. This measure, hereafter referred to as occurrence probability, is closely linked to persistence of a population, local range size and local species richness when accounting for species identity. Although occurrence probability is less information-rich and less-sensitive measure than abundance, it is more reliable and robust measure of population change when survey area is limited. We tested the following two hypotheses: (1) ungulate grazing decreases plant species occurrence, and (2) decrease in occurrence caused by ungulate grazing becomes more pronounced with increasing canopy openness.

We defined resilience as “the capacity to recover to pre-disturbance abundance levels through recruitment” and resistance as “the capacity of extant populations to survive disturbance through persistence”^32^. In many previous researches of population dynamics, presence of seeds within seed bank was treated as an indispensable condition for the population to recover to aboveground vegetation^32–36^. Based on the definition supported by aforementioned studies, we regarded seeds within a seed bank as a source of resilience for population, although seed bank can be regarded as a source of resistance for individuals.

## Results

We recorded 57 species within the plant survey, 939 seedlings representing 30 species in the germination experiment were distributed across five plots, with each plot consisting of eight quadrats measuring 1 × 1 m each. Among the species recorded, 36 found aboveground and 13 in the seed bank were classified as palatable. Canopy openness within the quadrats ranged 17–65%.

**Figure 2:**
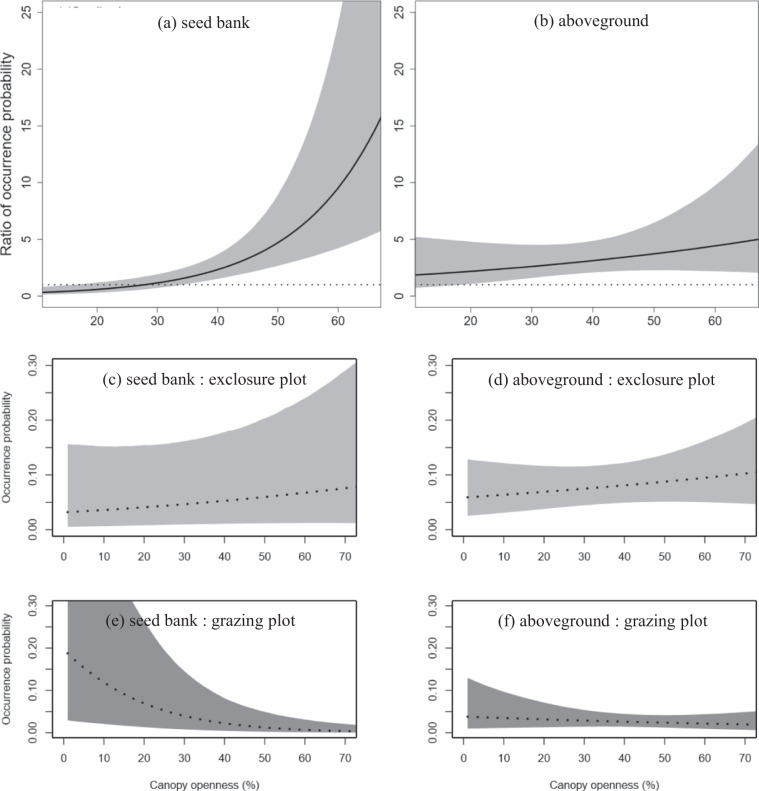
(**a**,**b**) Change in grazing impact severity represented by the ratio of plant species occurrence probability in grazing and exclosure plots along a gradient of canopy openness. Ratio > 1 indicates that the occurrence probability was larger in exclosure versus grazing plots. (**c–f**) The occurrence probability along gradient of canopy openness, lines show the mean of the ratio or that of occurrence probability and gray areas represent the 95% credible interval (CI).

The severity of deer grazing within the seed bank (defined as the ratio of occurrence probability of palatable species within exclosure plots compared to those within grazing plots) was significantly greater than one when canopy openness exceeded 34% (Fig. 2a) when controlling for species identity and spatial arrangement of the sampling quadrates. This trend was the result of a significant decrease in occurrence probability within grazing plots as canopy openness increased as well as a slight increase in occurrence probability within exclosure plots (Table 1). These data indicated that the occurrence probability within grazing plots is lower than that within the exclosures with high canopy openness. Furthermore, the occurrence probability within grazing plots was approximately one-tenth of that within exclosure plots when canopy openness was 65%, which was the maximum canopy openness in the studied quadrats (Fig. 2a). These results suggested that the interaction of two disturbances induced a synergistic impact on the occurrence probability of a plant species in the seed bank. Of note, the occurrence probability of a particular plant species in the seed bank also decreased significantly with increasing slope steepness (Table 1).

**Table 1:** The median and 95% credible interval (CI) of estimated coefficients within the models in which presence/absence of plant species in a quadrat is explained by exclosure/grazing plots and canopy openness.

	Median	2.5%	97.5%
**(a) In seed bank**			
**Intercept**	**−3.4**	**−5.3**	**−1.7**
**Exclosure**	**1.95**	**0.64**	**3.32**
Canopy openness (%)	0.01	**−**0.02	0.04
**Exclosure:openness**	**−0.07**	**−0.11**	**−0.04**
**Slope (°)**	**−0.061**	**−0.11**	**−0.018**
**(b) Aboveground**			
**Intercept**	**−2.5**	**−3.5**	**−1.6**
Exclosure	**−**0.47	**−**1.9	0.85
Canopy openness (%)	0.0086	**−**0.012	0.028
Exclosure:openness	**−**0.019	**−**0.052	0.014
Slope (°)	**−**0.015	**−**0.044	0.014

The significant variables are shown in bold text (p < 0.05)

In contrast to the aforementioned results, the occurrence probability for aboveground plant species was not significantly related to any predictor variable (Table 1). Similarly, to the seed bank, the occurrence probability tended to decrease in the grazing areas and increase in the exclosure plots with increasing canopy openness, although these differences were not statistically significant (Table 1). The ratio of occurrence probability within exclosure versus grazing plots was greater than 1, suggesting that ungulate grazing reduced plant occurrence. The occurrence probability increase was trivial within the range of canopy openness investigated (Fig. 2b).

## Discussion

Multiple disturbances may induce either synergistic or antagonistic impacts on ecosystems^5,11^, which are thought to result from the effects of supportive disturbances on the intensity, magnitude, or per capita strength of a main disturbance (described above as interaction chain and modification effects^4,9^). Here, we propose the existence of interaction exposure effects, in which a supportive disturbance expands or reduces the proportion of a population that is exposed to the main disturbance as yet another form of multiple disturbance interaction. In particular, we show that an increase in canopy openness increased the severity of deer grazing on the examined seed banks (Fig. 2). Given that canopy gaps often facilitate germination within forests^28,29^, this increased grazing severity could be attributed to gap-enhanced seedling emergence, thereby decreasing the number of seeds reserved in the soil bank.

Many plant populations remain resilient to disturbance(s) via retention of some portion of their seeds in the seed bank^25,37^, and thereby help to prevent exposure of the entirety of a population to disturbance exposure. It is important to consider the effects of disturbance interactions on population resilience not only for appropriate ecosystem management but also to increase understanding of long-term persistence of populations and communities^17,38^.

Current resilience of populations to disturbances can dramatically influence future populations^39,40^. Within interaction exposure and modification effects, a supportive disturbance alters the effect(s) of main disturbances on a population without modifying the strength and extent of the main disturbance. These two effects differ in their impacts on population resilience: interaction exposure effects reduce population resilience, whereas interaction modification effects do not necessarily affect resilience^4,19^. Therefore, disturbance interactions affecting population resilience that have previously been treated as interaction modifications should instead be considered as interaction exposure effects in order to clarify the synergistic effects of multiple disturbances.

The key to distinguishing interaction exposure from interaction modification effects is determining whether or not a supportive disturbance changes the ratio of exposed to unexposed individuals within a species. In addition to the example we provide here, additional reports of interaction effects that have been regarded as interaction modification could be better classified as interaction exposure effects. For instance, Doherty *et al*.^8^ suggested that anthropogenic disturbances increased predation of native mammals by invasive predators via a reduction in the number of available shelters. Although this has been classified as a case of interaction modification effects, a more suitable interpretation may be to name this as interaction exposure effects, in doing so this would prevent an overlooking of the decrease in the recovery source for native mammals from the pressure of predation and consequently aid in measurement of population resilience to disturbances. This concept of interaction exposure effects could also be applied to hypoxia within ocean and fisheries, in which it limits areas available for fish near to the shore and consequently increases the susceptibility of fish to fishing pressures^6^. Interaction exposure effects could affect populations not only cumulatively but also antagonistically. In regions where ungulates are not highly abundant, blown-down woods could protect seedlings from ungulate grazing through creation of a natural fence^14^. In such a case, wind would reduce a portion of the population exposed to ungulate grazing, and thereby would be better treated as an antagonistic case of interaction exposure effects. In the future, climate change and expanding human development pressures will increase the frequency of disturbance interactions^5,41,42^. Therefore, interaction exposure effects between anthropogenic and natural disturbances would be expected to impact populations, communities, and ecosystems more frequently. Some reptiles climb trees to escape from floods^43^, and certain insects dig holes in river beds to withstand drought^44^; species adopting these avoidance strategies would be compromised by rapidly expanding habitat modification due to anthropogenic development (e.g., logging and agricultural land expansions wiping out tall trees, and covering of stream beds with concrete)^5^. Interaction exposure effects are an alternative and important form of interaction occurring in various ecosystems, although further studies including combinations of disturbances and larger datasets are needed to assess their prevalence and importance. Also, verification of the existence of interaction exposure effect is needed at community level to expand applicability of the concept, and to make the concept acceptable for researchers who consider resilience as a community-level propertiy^45–48^.

We showed that the occurrence probability of plant species within the seed bank was lower in grazing versus exclosure plots when canopy openness was high (Fig. 2a). If ungulates showed a preference for grazing under canopy gaps as opposed to a closed canopy, the observed pattern would be explained by interaction chain effects^4^, wherein the effects of a supportive disturbance impact the strength of main disturbances. However, we found that in the study area foraging ungulates did not appear to be biased toward gaps either before or during the survey period for the following reasons: first, grazing had considerably decreased the quantity of palatable plants throughout the island and ungulates had nothing left forage except fallen leaves^49,50^; second, these animals did not appear to stay within particular grazing patches (i.e., under canopy gaps), likely because grazing patch selectivity decreases when ungulate density is high^51^. The empirical finding - i.e., interaction of gap creation and ungulate grazing decreasing population resilience - of this study was classified to be interaction exposure effects under the assumption that canopy gaps were created with similar timing. This assumption is required in order to omit the influence of canopy gap duration on the seed bank, as a decrease in seeds would be more severe under canopy gaps that were created earlier compared to those created recently, when gap size is equivalent. Most canopy gaps in this area, however, were likely to be created by the historically large typhoon occurring in the Hokkaido prefecture in 2004^52,53^. For interpretation of the interactive effects of gap creation and ungulate grazing on plant populations in other areas, attention to the difference in periods in which gaps were created across the focal area would be necessary. Gap creation and ungulate grazing are major disturbances in forest ecosystems^41,54^; hence, depletion of the seed bank owing to interaction exposure effects from these disturbances could occur in other forest ecosystems where ungulate density is high. Furthermore, the impact of these effects would increase in plant communities composed largely of palatable species. Further surveys in other areas with varying ungulate density or species composition could confirm the magnitude of interaction exposure effects on communities as a whole. Although we adopted an orthodox protocol^24,40,55^ with a wide temperature range for our germination experiment, additional germination experiments utilizing various germination cues would assist us in application of our results to other scenarios^56^.

In contrast, the occurrence probability of aboveground plants was stable along the gradient of canopy openness, regardless of grazing (Table 1). This result is partly supported by a previous finding by Suzuki & Ito^57^, who showed that terrestrial plant species richness is influenced to a greater degree by ungulate grazing versus canopy gap creation in areas of high ungulate density. Even if the occurrence probably in the seed bank under canopy gaps is less than that under the closed canopy, the occurrence of aboveground plants would remain higher under canopy gaps. This notion is supported by findings that showed canopy gaps provide more suitable conditions for germination than a closed canopy^28–30^. Multiple disturbances with interaction exposure effects may change components of populations that are more difficult to observe early compared to those that are easily observed.

To verify interaction exposure effects on our field data, the linkage between occurrences at aboveground and at seed bank needs to be confirmed. We, however, were unable to empirically confirm the linkage because of the limited number of observed species, and of species shared between seed bank and aboveground (Supplemental dataset). However, this lack of empirical verification should not be critical in confirming the existence of interaction exposure effect. Myriad studies have showed difference in species composition between aboveground vegetation and seedbank^45,56^, while the difference could be largely attributed to the difference in sampled area on vegetation and seedbank^40^. Moreover, Toräng *et al*.^35^ and Sletvold *et al*.^36^ clearly stated that seeds in seedbank is one of the most important factors for the population to recover to aboveground after disturbance. Therefore, the existence of the linkage between seed bank and aboveground is warranted in the context of population, and thus we considered that the effects interaction exposure effects was verified.

In conclusion, we proposed the concept of interaction exposure effects as a novel form of disturbance interaction using a case study of how these effects occur within a forest ecosystem. When multiple disturbances occur and exert interaction effects, these disturbances induce synergistic impacts on populations as interaction exposure effects impact a proportion of the population that could otherwise escape from the disturbance. To fully understand the impacts of co-occurring multiple disturbances, it is essential to determine the extent of the population exposed to disturbances.

## Materials and Methods

Our study area was Nakajima Island (4.84 km^2^) situated in the middle of Lake Toya, Hokkaido, northern Japan (42.5°N, 140.8°E). The climate is cold temperate, with mean annual precipitation and temperature being 518.0 mm and 8.7 °C, respectively. The mean monthly temperature ranges from −3.8 °C to 21.7 °C (data from the climatological observatory of Muroran). The snow-free period extends from April to November, whereas the growing season extends mainly from June to October. The majority of the island is covered by natural broad-leaved forests composed mainly of *Acer pictum* Thunb. subsp. *momo* (Maxim.) H. Ohashi and *Tilia japonica* (Miq.) Shimonk var. *japonica*^50^. Canopy gaps, created mainly by natural disturbance such as typhoons, are distributed sporadically along the forest canopy. The large ungulate *Cervus nippon yesoensis* Heude (mean withers height 1.5 m, capable of foraging plants up to 2 m in height) was introduced between 1957 and 1966 and inhabits the entire island. The understory vegetation experienced overgrazing for >30 years by ungulates, with a density of 21–91 individuals/km^2^ up to 2012^58^, which was subsequently reduced to 12 individuals/km^2^ after 2012^58^; however, this density is still above the sustainable level for growth and reproduction of the understory^59^. In 2004, several exclosures (approximately 30 × 30 m) were set up on the island.

### Survey design

We selected five exclosures varying in canopy openness and five adjacent installed grazing plots for the field survey. We divided each exclosure and grazing plot into four squared subplots, and placed a 1 × 1 m quadrat at the center of each subplot (40 quadrats in total). The soil moisture content in the quadrats ranged 41.2–54.9%. We conducted a vegetation survey and soil sampling as well as measurement of environmental conditions in all quadrats.

During the vegetation survey conducted in July 2015 we identified and recorded all vascular plant species with a height <2 m in each quadrat, including seedlings. We set the upper limit of plant height to 2 m based on the maximum reach for grazing and browsing of *Cervus nippon yesoensis* Heude^59,60^.

We randomly sampled five soil cores using a sampling cylinder 5.0 cm in diameter and 5.1 cm in height from the surface of each quadrat in July 2015 to examine the persistent seed bank following the peak germination time for the majority of plant species in the study area. Soil samples were protected from sunlight after collection and transferred to the laboratory.

Soil samples were enclosed in plastic bags and stored under dark and cold conditions (i.e., 5 °C) for 3 months. We initiated the germination experiment during October 2015 using half of each sample (50 ml) after thorough mixing according to a previous study^24^. The soil was deposited in thin layers (≤1 cm) on 2 cm-thick vermiculite in plastic packs (115 × 85 × 45 mm) containing several holes on the bottom. We placed the packs in a greenhouse under a light/dark cycle of 12/12 h. The temperature during the light cycle was set to 25 °C, which is the highest mean temperature during August in the region, whereas the dark cycle temperature was set to 13 °C, i.e., the mean lowest temperature for June. The soil was kept constantly moist. To avoid light bias, we rearranged the packs every two weeks. We removed emerging seedlings after identification. We maintained plant growth for an additional two weeks when seedlings were too small to allow for identification. The experiment was continued until no additional germination occurred during two weeks, with the experiment having continued for 3 months.

### Environmental factor survey

We measured the environmental conditions of each quadrat during October 2015, before defoliation initiation. To measure canopy openness for each quadrat, we captured hemispheric photographs facing vertically upward using a digital camera (COOLPIX P6000, Nikon, Minato, Tokyo, Japan) with a fisheye lens (UWC-1689, Fit, Simosuwa, Nagano, Japan). We captured the photographs from 2 m above the center of each quadrat, and calculated canopy openness from the photographs using CanopOn2 ver.2.0.0 (http://takenaka-akio.org/etc/canopon2/). We measured slope gradients of ground surfaces using a clinometer at the center and four corners of each quadrat, and used the mean of these five measurements for analysis (see Table S1 for environmental conditions summary).

### Statistical analysis

We conducted two-step analyses to examine whether canopy openness changes the relationship between ungulate grazing and the occurrence probability of plant species. In the first step, we analyzed the relationship between plant species occurrence and environmental factors, i.e., canopy openness, presence/absence of ungulates, and slope gradient. Then we compared the occurrence probability of a plant species in an exclosure versus grazing plot along every 1% of the canopy openness gradient. We used the presence/absence of terrestrial plants and seeds in the bank as response variables. Ungulates preferentially graze on palatable species when those species are present^61,62^, thus we confined our analyses to only palatable species. We classified each plant species into palatable and unpalatable species based on the work of Hashimoto & Fujiki (2014)^63^.

Initially we investigated whether the presence/absence of ungulate grazing and canopy openness and their interaction affected the occurrence probability of any particular plant species. Using data on those species classified as palatable (13 in seed bank, 36 aboveground), we developed a Bayesian generalized liner mixed model in which the presence/absence of each species in a quadrat is explained by the presence/absence of ungulates, canopy openness, and their interaction as well as slope gradient. We added slope gradients to the model in order to account for the tendency of seeds to be washed off of steep slopes. We included IDs on pairs of exclosure and grazing plots (site ID), and species ID as random effects in the model to account for pseudo-replication, spatial arrangement of survey sites and species identity. Non-informative distributions with means of zero and variances of 10,000 were used as the prior distribution of fixed effects. We used normal distributions with means of zero and variances of *r* as the prior distributions for random effects; *r* represents hyperparameters with reciprocals following a uniform non-informative and non-negative distribution from 0 to 1.0 × 10^9^. We used a logit link function and a binomial error distribution. The posterior distributions of the model parameters were estimated by the Markov chain Monte Carlo (MCMC) method using WinBUGS^64^ (ver. 1.4.3). We ran the MCMC sampling for 30,000 iterations of three chains with a thinning interval of three iterations; iteration was set to 30,000 based on visual assessment of convergence of the three chains; we discarded the first 3,000 samples as burn-in. The convergence of the chains was judged by whether the Gelman–Rubin statistic was <1.1^65^.

To then investigate change in the degree of the effect of ungulate grazing on plant species along the gradient of canopy openness, we assessed the severity of deer grazing - the ratio of plant species occurrence probability in exclosure versus grazing plots - along every 1% of canopy openness by following the three processes outlined below. First, we substituted a set of coefficients obtained from the MCMC samples into the parameter of the model constructed in the first step. We used a value of zero for the slope gradient, with random effect of the site and that of species ID not being considered. Secondly, we calculated the occurrence probability in the exclosure and grazing plots along every 1% of the canopy openness gradient by increasing the openness from 17–65% in order to calculate prediction values. Finally, we calculated the ratio of prediction values in exclosure versus grazing plots. We applied these processes to 9,000 MCMC samples obtained in the first step, and calculated the 95% credible interval (CI) of the ratio. When the occurrence probability within exclosure plots was significantly larger than that within grazing plots, the 95% CI of the ratio was >1. We used Bayesian methods rather than traditional analysis in the first step in order to calculate the 95% CI of deer grazing severity using MCMC samples in the second step. All analyses, expect for Bayesian inference, were performed with the statistical software package R3.1.1^66^. A value of p < 0.05 was considered statistically significant.

## Supplementary information

Supplementary Information.

Supplementary Dataset.
