# Supplementary material for: Interaction exposure effects of multiple disturbances: plant population resilience to ungulate grazing is reduced by creation of canopy gaps
Source: Sci Rep. 2020 Feb 4;10:1802. doi: 10.1038/s41598-020-58672-6 (PMC7000668; doi:10.1038/s41598-020-58672-6)
Supplement: Supplementary file 1 — Supplementary Information. [file 41598_2020_58672_MOESM1_ESM.pdf]

Supplemental Information

Interaction exposure effects of multiple disturbances: plant population resilience to ungulate grazing is reduced by creation of canopy gaps

Yushin Shinoda* and MunemitsuAkasaka

Table S1. The mean, SD, minimum and maximum of the environmental conditions on survey quadrats

	Mean	SD	Minimum	Maximum
canopy openness (%)	37.5	13.5	17.4	65.0
slope (°)	15.7	9.4	2.0	46.8

Table S2. Occurrence probability for each values of exclosure, canopy openness and slope. The columm of exclosure shows exclosure (1) or grazing plot (0)

Exclosure	Canopy openness	Slope	occurrence probability
1	15	0	0.037
0	15	0	0.087
1	65	0	0.060
0	65	0	0.005
1	15	15	0.015
0	15	15	0.037
1	65	15	0.025
0	65	15	0.002
